# Ultrasonographic adrenal gland changes in dogs with Cushing’s syndrome with a low-dose dexamethasone suppression test result consistent with partial suppression or escape pattern

**DOI:** 10.3389/fvets.2024.1477208

**Published:** 2024-12-04

**Authors:** Matthias Mayr, Vera Geisen, Stefan Unterer, Astrid Wehner

**Affiliations:** ^1^Clinic of Small Animal Medicine, LMU Munich, Munich, Germany; ^2^Clinic for Small Animal Internal Medicine, Vetsuisse Faculty, Zurich, Switzerland

**Keywords:** hyperadrenocorticism, ACTH-dependent, ACTH-independent, adrenal-dependent, pituitary-dependent, adrenal width

## Abstract

**Background:**

Cushing’s syndrome (CS) in dogs is mainly caused by pituitary-dependent (PDH) or adrenal-dependent (ADH) hypercortisolism. Result of the low-dose-dexamethasone suppression test (LDDST) with partial suppression (PSP) or escape pattern (EP) are indicative of PDH. No data concerning the ultrasonographic characteristics of the adrenal glands from dogs with these patterns exists.

**Objective:**

To describe ultrasonographic appearance of adrenal glands in dogs with CS, with a LDDS test result consistent with PSP or EP.

**Animals:**

Forty-nine client owned dogs with a diagnosis CS with a PSP or EP in the LDDST.

**Methods:**

Retrospective evaluation of ultrasonographic adrenal gland size, shape and echogenicity. The dorsoventral thickness ratio (DVTR) and dorsoventral thickness difference ratio (DVTDR) was calculated.

**Results:**

PSP and EP occurred in 42.9 and 57.1% of all LDDST. The median maximum width of the left adrenal gland was 0.71 cm (IQR, 0.24 cm) and of the right 0.75 cm (IQR, 0.19 cm) in all dogs and there was no difference between both patterns. There was a significant correlation between adrenal gland width and weight (left adrenal gland *p* = 0.002, right adrenal gland *p* = 0.017). In 9/49 dogs (18.4%), an adrenal asymmetry with a DVTDR >0.3 was present. In 7 of these 9 dogs, follow-up was available indicating presence of PDH.

**Conclusions and clinical importance:**

Based on follow up, PSP and EP of the LDDST were very supportive of PDH. Bilaterally symmetric adrenomegaly is a characteristic finding in dogs with PDH, however the size of adrenal gland width in this cohort was smaller than previously reported. Adrenal asymmetry was noted in approximately 20%.

## Introduction

Naturally-occurring Cushing’s syndrome (CS), one of the most common endocrinopathies in dogs (1), affects mainly middle-aged and older dogs (1–5) and is common in small-breed dogs (1, 2, 5–8).

A distinction is made between ACTH-dependent and ACTH-independent hypercortisolism (9, 10). A further classification of ACTH-dependent disease can be made into pituitary-dependent hypercortisolism (PDH) and hypercortisolism with ectopic ACTH-secretion. The latter is usually caused by malignant tumors of neuroendocrine origin and is very rare (11). Adrenocorticotropin-independent disease can be classified into adrenal-dependent hypercortisolism (ADH) and hypercortisolism due to aberrant adrenal receptor expression. In the latter, food ingestion induces gastric inhibitory polypeptide which binds to receptors in the adrenal glands and causes excess cortisol secretion. This condition is also very rare (12, 13).

In PDH, both adrenal glands are stimulated to synthesize cortisol in the zona reticularis due to increased secretion of ACTH from the pituitary gland, resulting in bilateral adrenocortical hyperplasia and hypertrophy (9, 14). Tumors in the anterior or middle lobe of the pituitary gland are responsible for the increased synthesis of ACTH (15). PDH is the most common cause of naturally occurring CS. In older studies, 80 to 85% of patients were classified to have PDH (6, 16).

Depending on the study, between 63.4 and 83.3% of dogs with histologically confirmed ADH, were affected by functional adrenal carcinomas, and between 16.7 and 36.6% by functional adrenal adenomas (3, 17). These results may contain some bias, as necropsy was primarily performed in dogs with aggressive tumors (3). In another study of 52 dogs that underwent adrenalectomy, 48.2% had adenomas, 34.6% had adenocarcinomas, 13.4% had pheochromocytomas, and 3.8% had myelolipomas (18). Functional adrenal tumors occurred unilaterally in the majority of dogs (10). The low-dose dexamethasone suppression test (LDDST) examines the sensitivity of the hypothalamic–pituitary–adrenal axis to negative glucocorticoid feedback. The LDDST has been reported to have a high sensitivity for diagnosing CS, ranging from 85 to 97% (16, 19, 20). If the 8-h (h) cortisol value of the LDDST is diagnostic for CS, a partial suppression pattern (PSP), defined as 4- and 8-h cortisol value >1 μg/dl but either <50% of the basal value, and an escape pattern (EP), defined as 8-h cortisol value >1 μg/dl and the 4-h value <1 μg/dl, have only been described in dogs with PDH (20, 21). However, the later test result could also indicate a false positive test outcome (20, 21). Patients with ADH do not show signs of suppression (LDDST with 4- and 8-h cortisol value >1 μg/dl and both >50% of the basal values). However, a lack of suppression can also occur in PDH, and differentiation was only possible in approximately 55% of cases using the LDDST pattern (20, 22, 23).

Ultrasound of the adrenal glands is a frequently used diagnostic tool for CS. Changes in shape, size, and echogenicity of both adrenal glands may be observed. In practice, the maximum dorsoventral width is the most used parameter to assess adrenal gland size, as it demonstrated the least variability between different investigators (24, 25). A maximum dorsoventral width of the left adrenal greater than 7.4 mm was considered consistent with adrenal enlargement in one study (26). However, other studies showed that the size of the adrenal glands can vary depending on breed and body size (27, 28). Bilateral adrenal enlargement or least uniform adrenal glands are expected in dogs with PDH and adrenal asymmetry of the adrenal glands in dogs with ADH (29). However, asymmetry between the adrenal glands does not always indicate adrenal neoplasia as nodular hyperplasia or myelolipoma formation has been documented in PDH (10). If the adrenal glands were asymmetric, a width of the smaller adrenal gland less than 5 mm is suggestive for ADH according to one study (10). Concurrent PDH and ADH occurred in 1.1 to 5% of dogs with CS (30, 31).

The aim of this retrospective study was to describe the ultrasonographic adrenal gland appearance in dogs with CS, where the LDDST showed a PSP or EP.

## Materials and methods

### Case selection

Between 2006 and 2021 dogs newly diagnosed with CS at the Clinic of Small Animal Medicine, Ludwig Maximilian University Munich, were identified from medical records.

Dogs were included, if the LDDST was diagnostic for CS based on an 8 h concentration > 1.0 μg/dl and the cortisol pattern was consistent with PSP or EP (cortisol value after 4 and/or 8 h < 50% of basal value or after 4 h ≤ 1.0 μg/dl). Additionally, all dogs were required to meet at least one of the following criteria:

1. Presence of three or more common clinical signs. 2. Presence of at least two common clinical signs and at least one common laboratory abnormality. 3. Presence of one or more common clinical signs, two or more common laboratory abnormalities, and a positive response to treatment. 4. Presence of at least one common clinical sign, one less common clinical sign, and one common laboratory abnormalities. 5. Presence of at least one common clinical sign and at least four common laboratory abnormalities. For details on the definition of clinical signs and laboratory abnormalities, refer to Table 1.

**Table 1:** Definition of clinical signs and laboratory abnormalities.

Common clinical signs and physical examination findings	Less common clinical signs and physical examination findings	Common laboratory abnormalities	Positive response to treatment with trilostane
Polyuria/polydipsia	Lethargy/exercise intolerance	Elevated alkaline phosphatase	Improvement in clinical signs
Polyphagia	Hyperpigmentation	Elevated alanine aminotransferase	
Increased panting	Insulin-resistant diabetes mellitus	Hypercholesterolemia	
Abdominal distension		Neutrophilia and/or lymphopenia	
Endocrine alopecia		Thrombocytosis	
Muscle weakness/muscle atrophy		Urine specific gravity <1.020	
Calcinosis cutis		Proteinuria	

Ultrasonographic examination of both adrenal glands must have been performed at the time of diagnosis before treatment was initiated. Follow-up data of dogs were also taken into account.

Dogs with another LDDST pattern (e.g., no suppression) were excluded. If only one of the two adrenal glands was visualized sonographically or if information on the width of the adrenal glands was not documented, dogs were also excluded.

### Adrenal function testing

For the LDDST, a venous catheter was placed in the antebrachii cephalic or lateral saphenous vein, and serum was collected to determine basal cortisol levels. Subsequently, 0.01 mg/kg bodyweight of dexamethasone (Hexadreson, MSD Tiergesundheit, Unterschleissheim, Germany) was administered intravenously, and the venous catheter was removed. After 4 and 8 h, blood was drawn for determination of subsequent cortisol levels.

During testing, dogs were hospitalized in a quiet kennel with free access to water. Only 1–2 short walks were allowed. If a dog was persistently restless, the test was discontinued. As recommended in the ACVIM Consensus Statement, dogs were not fed during the LDDST (23) but had received a meal before the test was started.

### Cortisol assays

Cortisol concentration was measured using a solid phase, competitive chemiluminescent enzyme immunoassay (IMMULITE 2000 Cortisol, Siemens Healthcare Diagnostics, Erlangen, Germany) validated for dogs (32, 33). Samples were analyzed by an external laboratory (IDEXX Laboratories, Ludwigsburg, Germany).

### Ultrasonographic examinations

Ultrasound examinations were performed by board-certified Diplomates in Internal Medicine [ECVIM-CA (Internal Medicine)]. All dogs were examined with minimal manual restrain in dorsal recumbence. Examinations performed before 2008 were undertaken with the GE healthcare Logiq 5, between 2008 and 2014 with the GE healthcare Logiq P6, and since 2014 with the GE healthcare Logiq E9 (GE Healthcare, Chicago, USA). A 7–12 MHz or 8.4–9.0 MHz vector transducer or a 6–10 MHz curvilinear transducer was used.

Adrenal glands were evaluated for shape (physiologic, nodular, mass), echogenicity (physiologic, hyperechogenic, inhomogeneous), size (maximum dorsoventral width of the cranial and caudal pole or overall maximal dorsoventral width), and other possible changes (e.g., thrombus or vascular invasion of the caudal vena cava). A hypoechogenic peanut shape of the left and a hypoechogenic bean shape of the right adrenal gland was considered as physiologic. Depending on the echogenicity, enlargement of only one of the two adrenal poles was usually addressed as nodular hyperplasia or myelolipoma formation, while an enlargement of the adrenal gland that did not allow differentiation between the cranial and caudal poles was defined as an adrenal mass.

As far as data on the width of both adrenal glands were available, the dorsoventral thickness ratio (DVTR) and dorsoventral thickness difference ratio (DVTDR) were calculated. DVTR is defined as the ratio between LDV (maximal dorsoventral thickness of the larger gland) and SDV (maximal dorsoventral thickness of the smaller gland; DVTR = LDV/SDV). If one of the poles of the one adrenal was larger than the other, the larger diameter was used in this study. DVTDR is defined by the difference between LDV and SDV with respect to the mean [DVTDR = 2 × (LDV − SDV)/(LDV + SDV)] (10). Significant adrenal asymmetry was defined as an upper threshold of the 95% confidence interval of DVTR and/or DVTDR for the study population.

### Data analysis

Data were analyzed using GraphPad Prism® 10 (Fa. GraphPad Software Inc., La Jolla, USA). All parameters were evaluated descriptively. In addition to the median, the interquartile range was also calculated. To compare the group of dogs with PSP with the group of dogs with EP, the Mann–Whitney U test was used. A multiple logistic regression analysis was conducted to examine the correlation between a specific suppression pattern (EP or PSP) and various variables, including weight, age, gender, adrenal gland size (maximum width of the right and left adrenal glands), the difference in maximum width between the left and right adrenal glands, DVTDR, and DVTR. To further explore whether categorical variables, such as different weight and age groups, influence adrenal gland size, the study population was divided into three weight groups (<10, 10–20, and > 20 kg) and two age groups (<10 years and >10 years). The Kruskal-Wallis test and Mann–Whitney U test were used for the analysis. Additionally, a Spearman correlation was performed to assess the relationship between adrenal gland size and both age and weight. A significance level of *α* = 0.05 was used for all tests.

## Results

**Figure 1:**
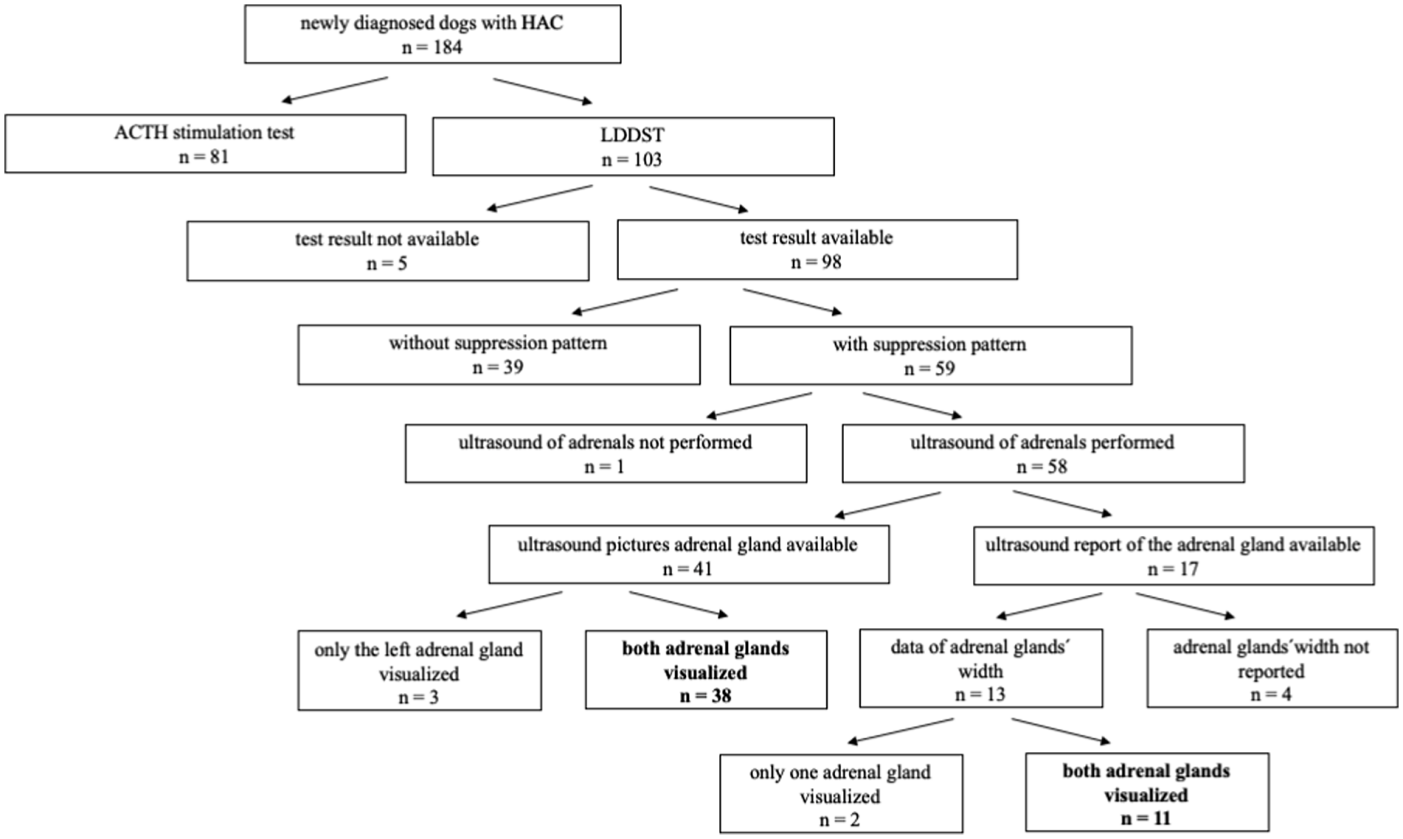
Flow diagram showing the selection of cases included in the study, bold print: both adrenal glands visualized. CS, Cushing’s syndrome; LDDST, low-dose dexamethasone suppression test.

In the analyzed period, 184 dogs were newly diagnosed with CS, of which 81 dogs were diagnosed by means of an ACTH stimulation test and 103 dogs by means of a LDDST. Of these, 5 LDDST results were not available to assess signs of suppression, because the 4 h value was missing in 2 dogs and the entire test results were not archived in 3 dogs. Of the 98 dogs, where the complete LDDST results were available, 59 dogs (60.2%) showed a PSP or EP and 39 dogs (39.8%) no suppression. In 58 dogs, an ultrasound of the adrenal glands was performed. In 53 dogs both adrenal glands could be visualized, whereas in 4 dogs only the left adrenal gland and in one dog only the right adrenal gland was identified. Of these 53 dogs, data of the adrenal glands’ width were not reported in four dogs. Thus, 49 dogs met the inclusion criteria (Figure 1). All of these 49 dogs showed signs and laboratory findings consistent with CS. For more detailed data, please refer to the Supplemental data (Supplementary Tables S1, S2). Additional imaging of the pituitary gland by computer tomography (CT) or magnetic resonance imaging (MRI) was available in four dogs. Two dogs had a pituitary macrotumor and two had a microtumor. Measurement of endogenous ACTH was available in one dog.

### Signalment of dogs with CS

Of the 49 dogs included, 30 dogs were female (61.2%) of which 21 were spayed and 19 dogs were male (38.8%) of which 11 were neutered. Age ranged between 7 and 16 years (median age 11 years). Body weight ranged between 3.8 and 39.0 kg (median weight 11.1 kg; Table 2). Dogs were mixed breed (*n* = 14; 28.6%) or purebred dogs (*n* = 35; 71.4%). The most common breeds were West Highland White Terrier (*n* = 6) and Yorkshire Terrier (*n* = 5; Table 3).

**Table 2:** Descriptive statistics for age, weight and sex.

Parameter	*n* = 49
Age (years)	Median: 11
	Range: 7–16
	IQR^1^: 3.0
Weight (kg)	Median 11.1
	Range: 3.8–39.0
	IQR^1^: 14.8
Gender	
Female [*n*, (%)]	30 (61.2)
Female spayed [*n*, (%)]	21 (70.0)
Male [*n*, (%)]	19 (38.8)
Male neutered [*n*, (%)]	11 (57.9)

1 IQR: interquartile range.

**Table 3:** Overview of dog breeds in this study.

Breed	*n* = 49
Mixed breed	14
West Highland White Terrier	6
Yorkshire Terrier	5
Jack Russel Terrier	4
Dalmatian, Dachshund, Labrador Retriever, Magyar Viszlar	2 each
Anglo-Français de petite vénerie, Australian Labradoodle, Bearded Collie, Bichon Frisé, Cairn Terrier, Cavalier King Charles Spaniel, English Cocker Spaniel, French Bulldog, Golden Retriever, Parson Russel Terrier, Pomeranian, Rhodesian Ridgeback	1 each

### Low-dose dexamethasone-suppression test results

Median and IQR of cortisol concentrations [basal, 4 h (h) and 8 h] are given in Table 4. Six dogs (12.3%) had an 8 h value >1.0 μg/dl and <1.4.

**Table 4:** Description of LDDST results with suppression criteria.

LDDST	0 h-value (*n* = 49)	4 h-value (*n* = 49)	8 h-value (*n* = 49)
Median (μg/dl; Range)	4.7 (1.8–12.5)	1.2 (0.1–4.8)	2.6 (1.1–10.0)
IQR^1^ (Q^2^_0,75_–Q_0,25_)	4.7 (7.8–3.1)	1.4 (2.0–0.6)	2.5 (4.0–1.5)

1 IQR: The interquartile range is a measure of dispersion and is the difference between the 3rd quartile and the 1st quartile.

The suppression pattern of the 49 dogs was classified as following:

- PSP (4 and 8 h >1 μg/dl but either <50% of t0): 28 dogs (57.1%)
- EP (8 h >1 μg/dl and 4 h <1 μg/dl): 21 dogs (42.9%; Table 5).

**Table 5:** Comparison of maximum width of the adrenal glands between dogs with partial suppression and escape pattern in dogs.

Parameter	Escape pattern (*n* = 21)	Partial suppresion pattern (*n* = 28)	*p*
Maximum width of left adrenal gland (cm)	Median: 0.70	Median: 0.76	0.7849
	Range: 0.39–1.19	Range: 0.40–2.3	
	IQR^1^: 0.24	IQR^1^: 0.24	
	(Q^2^_0.75_–Q^2^_0.25_): 0.84–0.60	(Q^2^_0.75_–Q^2^_0.25_): 0.83–0.59	
	*n* = 21	*n* = 28	
Maximum width of right adrenal gland (cm)	Median: 0.70	Median: 0.79	0.7926
	Range: 0.51–1.18	Range: 0.30–1.30	
	IQR^1^: 0.22	IQR^1^: 0.17	
	(Q^2^_0.75_–Q^2^_0.25_): 0.89–0.67	(Q^2^_0.75_–Q^2^_0.25_): 0.85–0.68	
	*n* = 21	*n* = 28	
Difference between max. width of left and right adrenal gland (cm)	Median: 0.15	Median: 0.13	0.7463
	Range: 0–0.54	Range: 0–2.0	
	IQR^1^: 0.13	IQR^1^: 0.22	
	(Q^2^_0.75_–Q^2^_0.25_): 0.18–0.05	(Q^2^_0.75_–Q^2^_0.25_): 0.25–0.03	
	*n* = 21	*n* = 28	
DVTR	Median: 1.17	Median: 1.18	0.9276
	Range: 1.0–2.3	Range: 1.0–7.67	
	IQR^1^: 0.24	IQR^1^: 0.31	
	(Q^2^_0.75_–Q^2^_0.25_): 1.31–1.07	(Q^2^_0.75_–Q^2^_0.25_): 1.35–1.04	
	*n* = 21	*n* = 28	
DVTDR	Median: 0.15	Median: 0.16	0.9276
	Range: 0–0.79	Range: 0–1.54	
	IQR^1^: 0.20	IQR^1^: 0.26	
	(Q^2^_0.75_–Q^2^_0.25_): 0.27–0.07	(Q^2^_0.75_–Q^2^_0.25_): 0.30–0.04	
	*n* = 21	*n* = 28	

1 IQR: interquartile, CI: confidence interval; DVTR: dorsoventral thickness difference ratio, DVTDR: dorsoventral thickness difference ratio.

### Ultrasonographic description of adrenal glands

**Figure 2:**
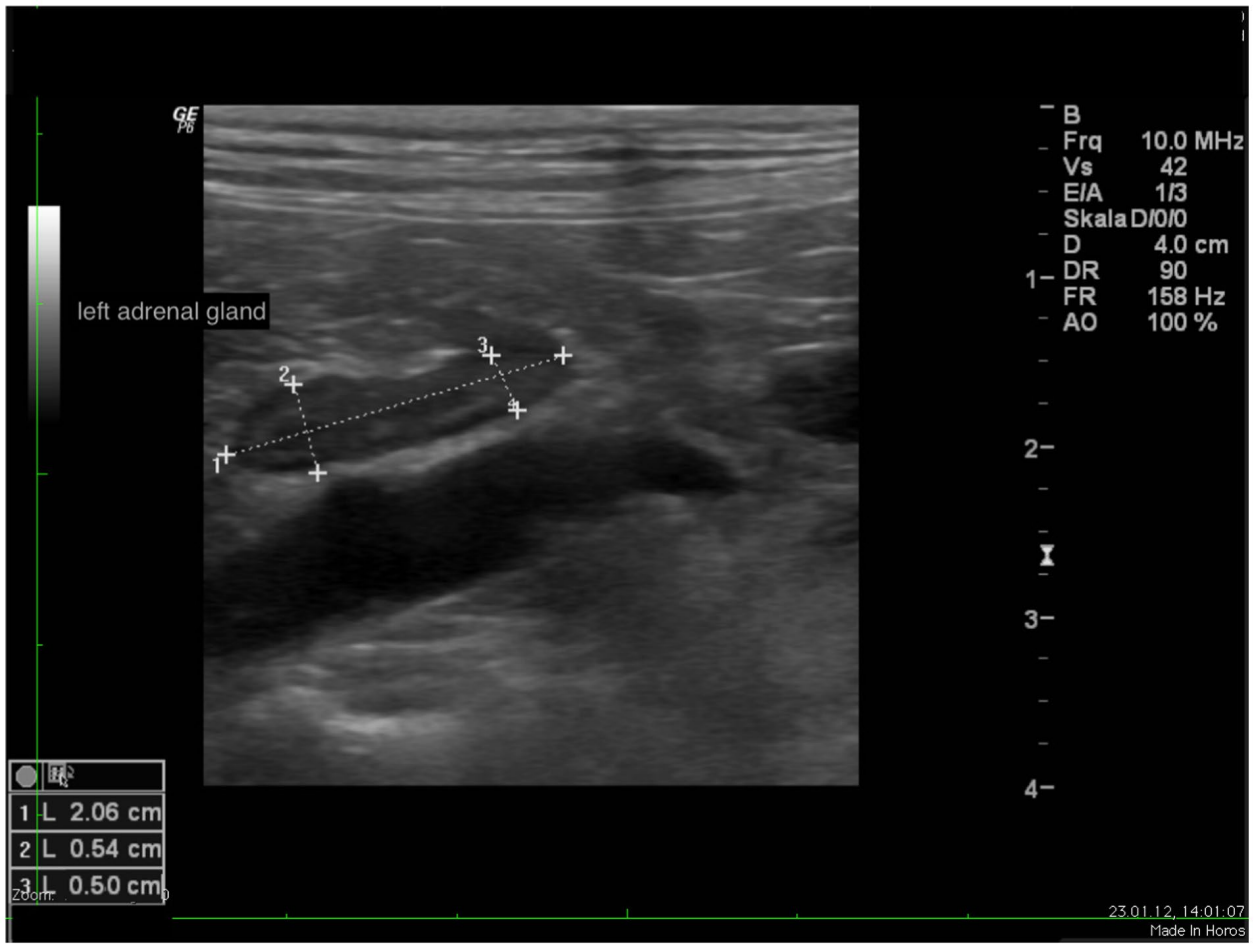
Ultrasound image of the left adrenal gland with measurement of the cranial and caudal pole (0.54 and 0.5 cm, respectively).

**Figure 3:**
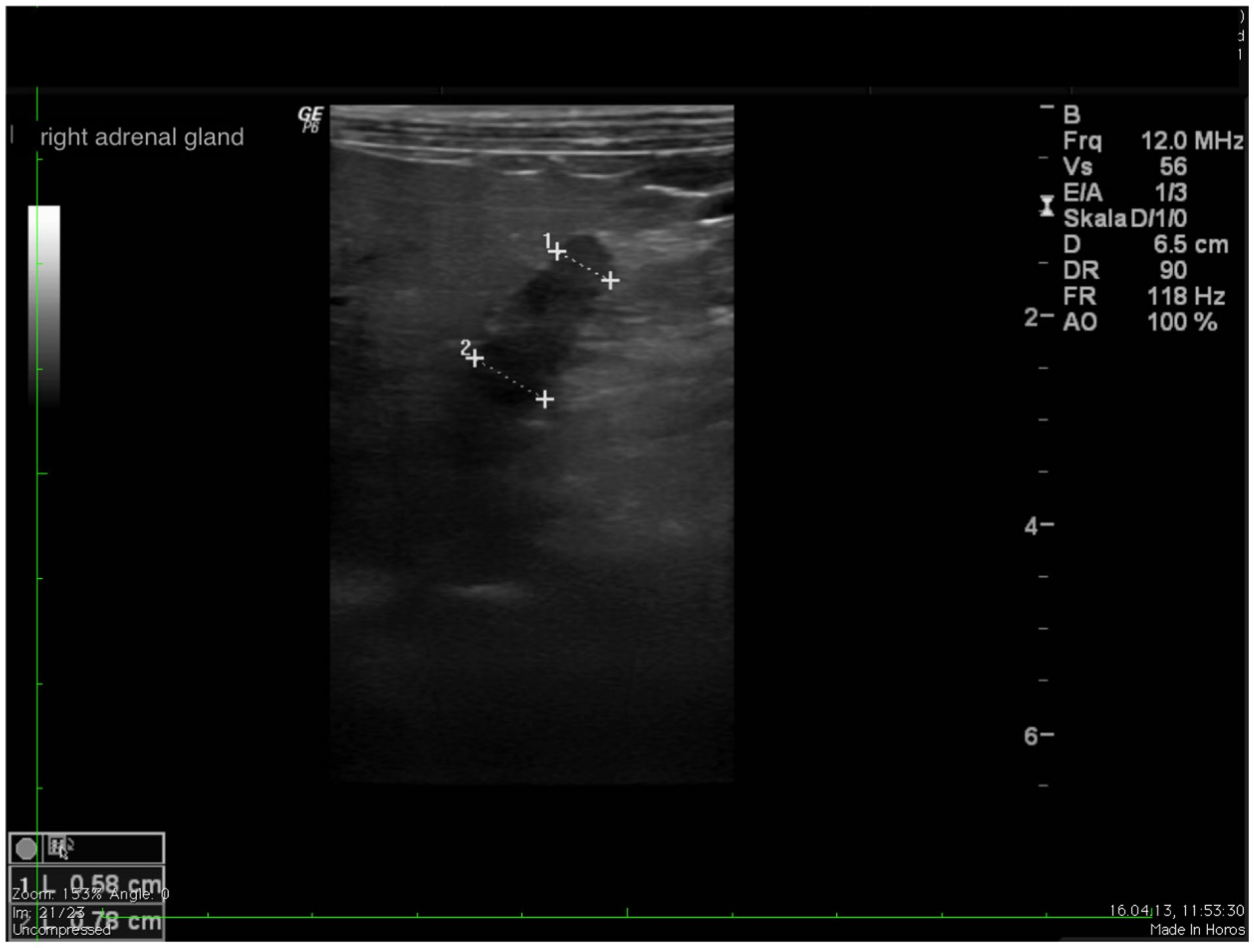
Ultrasound image right adrenal gland with a measurement of the cranial and caudal pole (0.78 and 0.58 cm, respectively).

**Figure 4:**
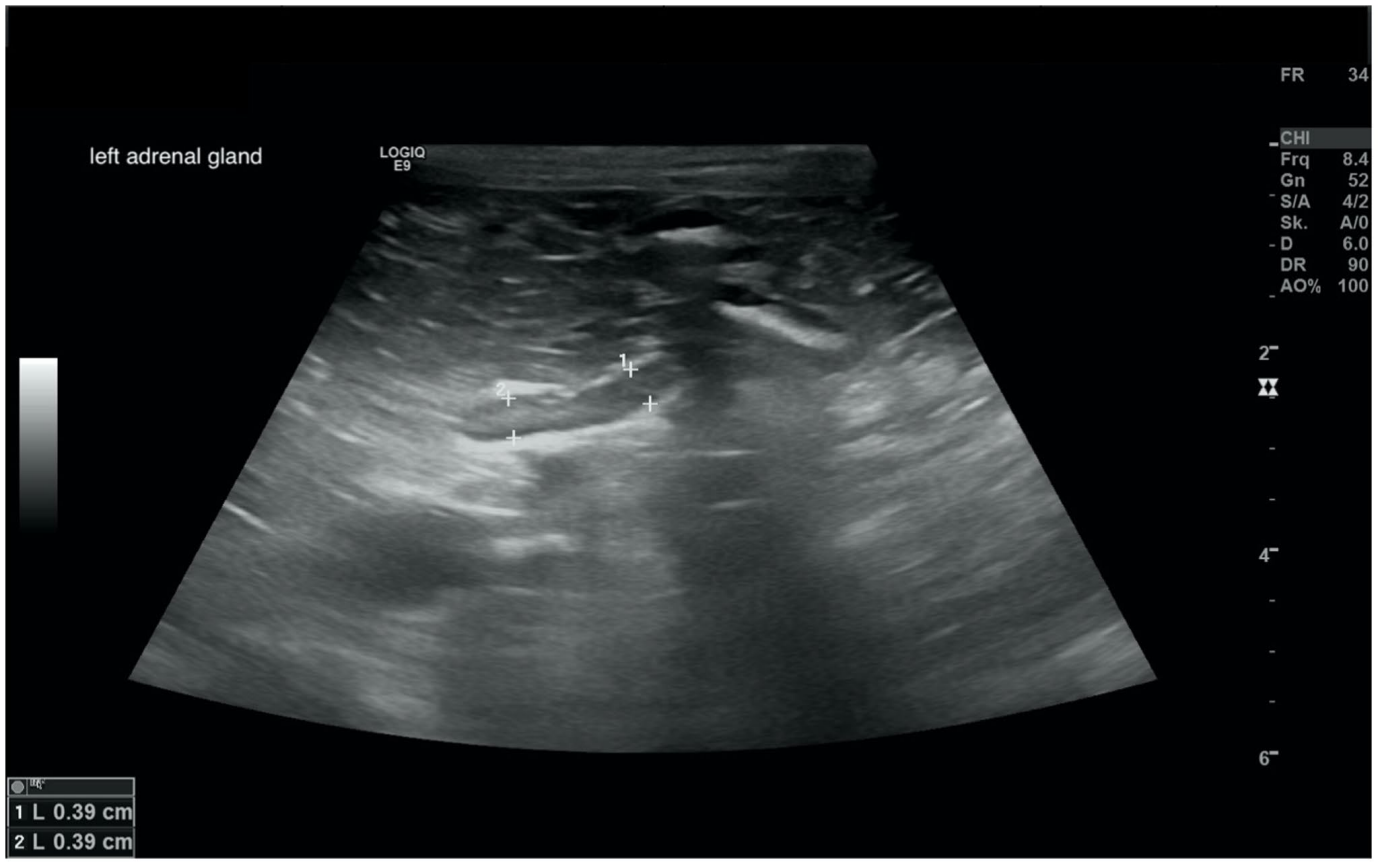
Ultrasound image of the smallest documented adrenal gland. This left adrenal gland measured 0.39 cm at the cranial and caudal pole, the right adrenal gland of this dog (not shown) measured 0.9 cm at cranial pole and 0.78 cm at the caudal pole; revaluation of the adrenals after 12 months revealed the following: cranial and caudal pole of the left adrenal gland 0.65 and 0.58 cm, cranial und caudal pole of the right adrenal gland 0.9 and 0.69 cm. Two years before the diagnosis of Cushing syndrome an ultrasound of the adrenal glands had been performed, revealing a left adrenal gland size of 0.48 cm and a right adrenal gland size of 0.53 cm.

In all dogs the maximum width of the adrenals was measured. The median maximum width of the left adrenal gland was 0.71 cm (IQR, 0.24 cm) and of the right adrenal gland was 0.75 cm (IQR, 0.19 cm). In a subset of dogs, measurements of the cranial and caudal adrenal poles were available. Thirty-eight measurements of the width of the cranial pole and 41 measurements of the width of the caudal pole of the left adrenal glands, and 38 measurements of the width of the cranial pole and 37 measurements of the caudal pole of the right adrenal glands were available. All detail data are shown in Table 6. The median difference between the two poles of the left adrenal gland (*n* = 37) was 0.11 cm (IQR, 0.12 cm) and of the right adrenal gland (*n* = 31) was 0.11 cm (IQR, 0.15 cm; Table 6). Figures 2, 3 show the measurement of a left and right adrenal gland. Figure 4 shows the image of the smallest documented adrenal gland.

**Table 6:** Description of the left and right adrenal glands.

Parameter	*n*	Median	Minimum	Maximum	IQR^1^
Cranial pole left adrenal gland (cm)	38	0.65	0.39	2.3	0.30
Caudal pole left adrenal gland (cm)	41	0.69	0.39	2.1	0.23
Difference between cranial and caudal pole left adrenal gland (cm)	37	0.11	0.0	0.66	0.12
Maximum width left adrenal gland (cm)	49	0.71	0.39	2.3	0.24
Cranial pole right adrenal gland (cm)	38	0.75	0.30	1.3	0.20
Caudal pole right adrenal gland (cm)	37	0.66	0.30	1.16	0.21
Difference between cranial and caudal pole right adrenal gland (cm)	31	0.11	0.0	0.72	0.15
Maximum width right adrenal gland (cm)	49	0.75	0.3	1.3	0.19

1 IQR: interquartile.

The difference in maximum width between the right and left adrenal glands was also calculated. For all 49 patients, the median width difference was 0.13 cm (IQR, 0.17 cm; Table 7).

**Table 7:** DVTR and DVTDR in all 49 dogs.

Parameter	Median	Minimum	Maximum	IQR^1^	Lower 95% CI	Upper 95% CI
Difference between max. width of left and right adrenal gland (cm)	0.13	0.0	2.0	0.17		
DVTR	1.17	1.0	7.7	0.27	1.11	1.66
DVTDR	0.16	0	1.54	0.22	0.15	0.31

1 IQR: interquartile range, CI: confidence interval, DVTR: dorsoventral thickness difference ratio, DVTDR: dorsoventral thickness difference ratio.

There were 11 dogs, where at least one adrenal gland pole was <0.5 cm wide. In none of the dogs, both adrenal glands or all of the four adrenal gland poles were < 0.5 cm wide. In 5 dogs, one gland (both poles) was <0.5 cm wide. One of those was a dog, where ADH was suspected. In 2 dogs, one pole of each adrenal gland was <0.5 cm wide. In 4 dogs, one pole of one adrenal gland was <0.5 cm wide. Five of the 11 dogs (4 with one adrenal gland width < 0.5 cm and 1 dog, where only one adrenal gland pole was smaller than 0.5 cm) are described in more detail in the cohort of dogs with DVDTR >0.3.

**Figure 5:**
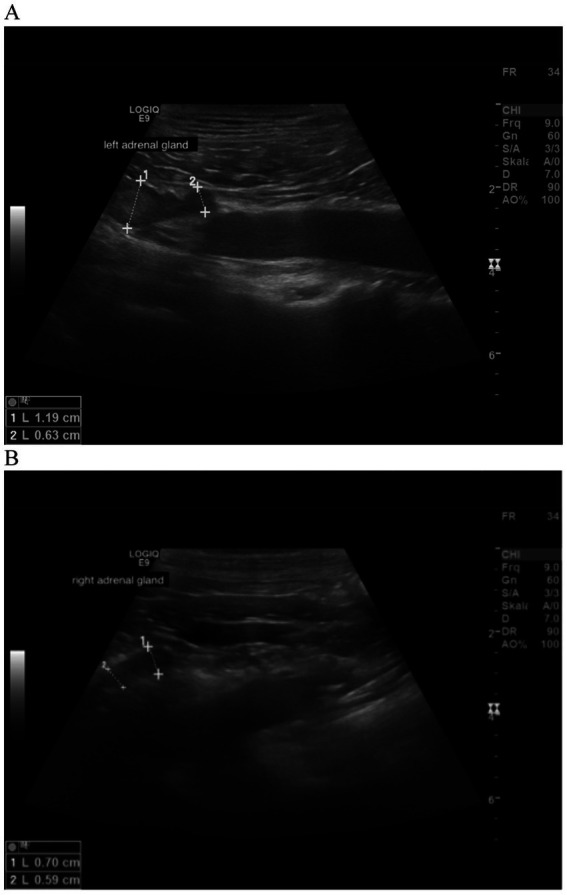
Ultrasound image of the left and right adrenal gland in a dog with adrenal asymmetry and a suspected myelolipoma. **(A)** In the cranial pole of the left adrenal gland, a round hyperechogenic lesion was present. Measurements were 1.19 cm at the cranial pole and 0.63 at the caudal pole. Revaluation of the adrenals was performed after 18 months. At this time, the cranial and caudal pole of the left adrenal gland measured 1.19 and 0.75 cm. **(B)** cranial and caudal pole of the right adrenal gland measuring 0.59 and 0.7 cm.

In 38 dogs, images of adrenal glands were archived and were available for evaluation of adrenal shape and echogenicity. Thirty-two (84.2%) had a physiological longitudinal oval shape. In 6 dogs (15.8%), nodular hyperplasia of one adrenal gland was present, whereas the contralateral adrenal gland had a physiological shape (Table 8). In two of these dogs a central lightening was present, which was addressed as adrenal myelolipoma (5.3%). Figure 5 shows the adrenal glands of a dog with suspected myelolipoma.

**Table 8:** Evaluation of the shape of the adrenal glands in all dogs with saved images (*n* = 38).

Finding	*n*	%
Both adrenal glands with physiological shape	32	84.2
One of the two adrenal glands with nodular hyperplasia	6	15.8

Regarding the echogenicity, the adrenal glands were both physiologically hypoechogenic in 32 dogs (84.2%), whereas in 5 dogs (13.2%) one of the two adrenals was hyperechogenic. In 1 dog (2.6%), one of the two adrenal glands had an inhomogeneous parenchyma. (Table 9). In none of the 38 dogs were thrombi formation or tumor invasion of vascular venous system identified.

**Table 9:** Evaluation of the echogenicity of the adrenal glands in all dogs with saved images (*n* = 38).

Finding	*n*	%
Both adrenal glands with physiologic echogenicity	32	84.2
One of the two adrenal glands hyperechogenic	5	13.2
Both adrenal glands with inhomogeneous parenchyma	1	2.6

The median DVTR was 1.17 (IQR = 0.27) and the median DVTDR was 0.16 (IQR = 0.22). The upper threshold of the 95% confidence interval for the DVTR was 1.7 and for DVTDR was 0.3 (Table 7). There were 9 dogs (18.4%) with a DVTDR >0.3 and 5 dogs with a DVTR >1.7. All 5 dogs with a DVTR >1.7 also had a DVTDR >0.3. The other 4 dogs with a DVTDR >0.3 had a DVTR ≤1.7, with a median DVTR of 1.6 (IQR = 0.25, range, 1.4–1.7; Table 10).

**Table 10:** Overview of 9 dogs with DVDTR >0.3.

	Dog	LDDST pattern	Imaging of pituitary	Sonographic follow up available	Left adrenal gland cranial pole (cm)	Left adrenal gland caudal pole (cm)	Right adrenal gland cranial pole (cm)	Right adrenal gland caudal pole (cm)	Maximum width left adrenal gland (cm)	Maximum width right adrenal (cm)	DVTR	DVTDR	Left adrenal gland shape	Left adrenal gland echogenicity	Right adrenal gland shape	Right adrenal gland echogenicity	Assessment
1	West Highland White Terrier	PSP		No follow-up	0.44	0.45	0.69	0.64	0.45	0.69	1.5	0.42	Physiologic	Hypoechogenic	Physiologic	Hypoechogenic	PDH
2	Anglo-Français de petite vénerie	PSP		No follow-up	2.3	2.1	0.3	0.3	2.3	0.3	7.7	1.54	°	°	°	°	ADH
3	Cairn Terrier	PSP		Yes	*	*	0.8	0.6	0.5	0.8	1.6	0.46	°	°	°	°	PDH
				✢													
4	Labrador Retriever	PSP		Yes	1.75	1.09	1.26		1.75	1.26	1.4	0.33	Nodular	Inhomogeneous	Physiologic	Inhomogeneous	PDH
				After 3 months	*	*	*	*	1.7	1.4	1.2	0.19					
5	West Highland White Terrier	EP		Yes	0.41	0.48	1.02	0.3	0.48	1.02	2.1	0.72	Physiologic	Hypoechogenic	Physiologic	Hypoechogenic	PDH
				After 12 months	0.54	0.57	1.13	0.6	0.57	1.13	2.0	0.66					
6	Labrador Retriever	EP		Yes	1.19	0.63	0.59	0.7	1.19	0.7	1.7	0.52	Nodular	Hyperechogenic^1^	Physilogic	Hypoechogenic	PDH + myelolipoma
				After 18 months	1.19	0.75	1.1	0.72	1.19	1.1	1.1	0.08					
7	Mixed Breed	EP		Yes	0.39	0.39	0.9	0.78	0.39	0.9	2.3	0.79	Physiologic	Hypoechogenic	Physiologic	Hypoechogenic	PDH
				After 12 months	0.65	0.58	0.9	0.69	0.58	0.9	1.6	0.43					
8	Cavalier King Charles Spaniel	PSP		No	0.54	0.54	0.99	0.74	0.54	0.99	1.8	0.59	Physiologic	Hypoechogenic	Nodular	Hypoechogenic	PDH
			MRI^2^														
9	Australian Labradoodle	PSP	CT^3^	Yes^4^	0.49	0.59	1.1	0.62	0.59	1.1	1.7	0.60	Physiologic	Hypoechogenic	Nodular	Hyperechogenic^2^	PDH + myelolipoma
				After 31 months	0.64	0.9	1.44	0.8	0.9	1.44	1.6	0.46					

°Images not saved, no information about shape and echogenicity. ✢after 18 month an ultrasound of the urinary tract was performed, and no adrenal mass was documented. ^*^Only total adrenal width reported. ^1^Hypoechogenic with a hyperechoic round area (suspected myelolipoma), ^2^MRI of the pituitary gland after 5 months: pituitary macrotumor. ^3^CT of the pituitary gland: pituitary microtumor, ^4^endogenous ACTH measurement 22.4 pg/ml (reference 9–67.7). ADH: adrenal-dependent hypercortisolism, CT: computer tomography, DVTR: dorsoventral thickness difference ratio, DVTDR: dorsoventral thickness difference ratio, EP: escape pattern, LDDST: low-dose-dexamethasone suppression test, MRI: magnetic resonance imaging, PSP: partial suppression pattern, PDH: pituitary-dependent hypercortisolism.

Of the 9 dogs with a DVTDR >0.3, 6 dogs had a PSP and 3 dog had an EP in the LDDST. Of the 5 dogs with a DVTDR >0.3, 3 dogs had a PSP and 2 dog had an EP in the LDDST.

Follow-up was available in 7 of the 9 dogs with a DVTDR >0.3. Follow-up ultrasonography of the adrenal gland was available in 5 dogs (after 3–18 months under therapy with trilostane).

**Figure 6:**
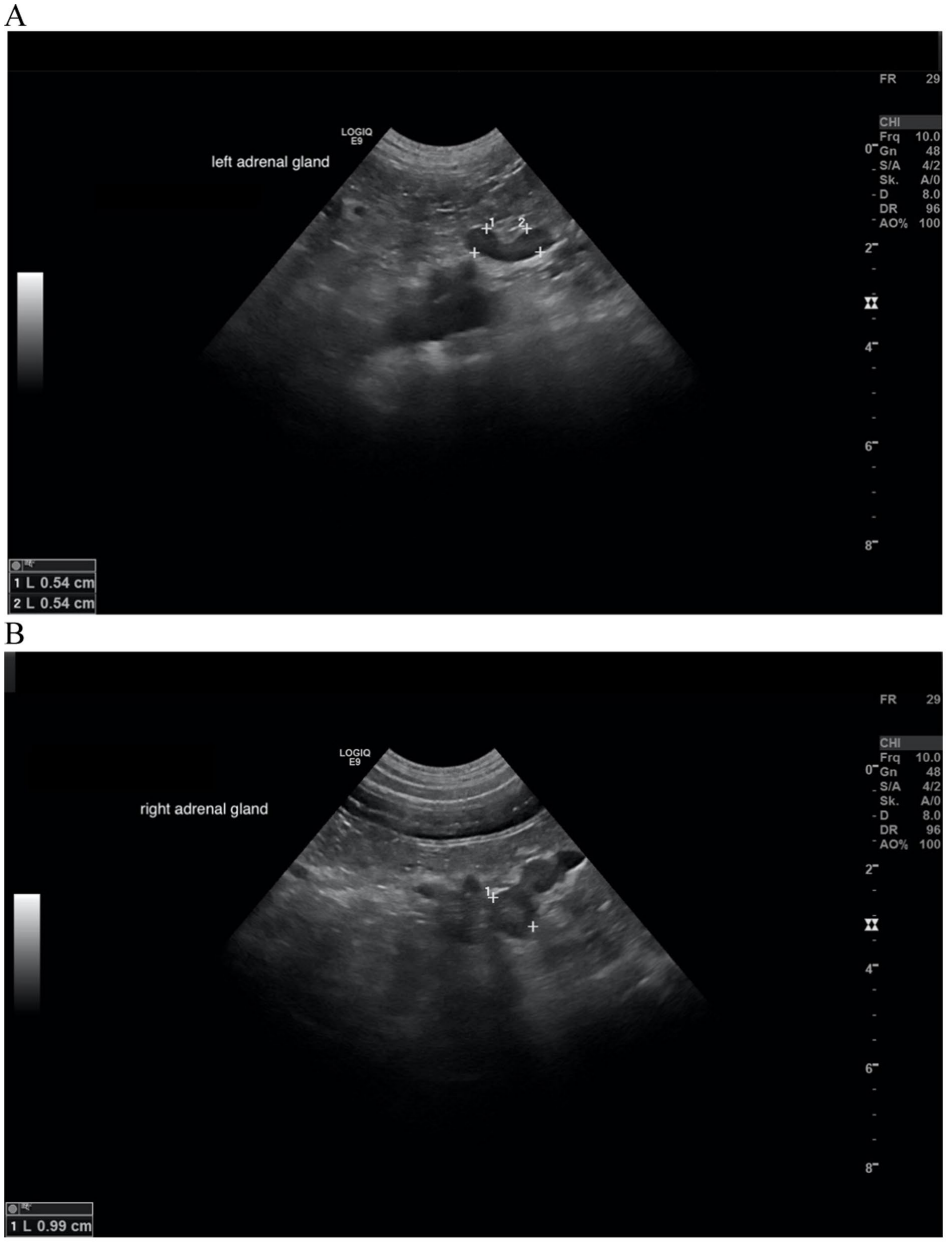
Ultrasound images of the left and right adrenal gland in a dog with adrenal asymmetry. **(A)** Cranial and caudal pole of the left adrenal gland measured both 0.54 cm in width. **(B)** cranial and caudal pole of the right adrenal gland measured 0.99 and 0.74 cm in width. In this dog, no reevaluation of the adrenal glands was performed. MRI had revealed a pituitary macrotumor.

In all 5 dogs with sonographic follow-up, the DVTDR decreased (0.79 → 0.43; 0.52 → 0.08; 0.60 → 0.46; 0.33 → 0.19; 0.72 → 0.66; Table 10). In the other two, one dog had an ultrasound of the urinary tract after 18 months, but the adrenal glands were not addressed in the report, and the other dog was diagnosed with a pituitary macrotumor by MRI after 5 months (Figure 6).

In 2 dogs, no follow-up was available, in one of those, one adrenal gland was 2.3 cm wide, whereas the contralateral adrenal gland was 0.3 cm wide. In this dog only the ultrasonographic report was available and images could not be reassessed. This was the only dog, where one adrenal gland was larger than 2.0 cm. In the other dog with DVTDR >0.3 without follow-up, the maximum width of the left adrenal gland was 0.45 cm and the maximal width of the right 0.69 cm. In this dog, both adrenal glands had a physiologic shape and echogenicity with no asymmetry between cranial and caudal pole of each adrenal gland.

If the dog with one adrenal gland >2.0 cm was excluded from the evaluation of the adrenal glands, the median maximum left adrenal gland width was 0.70 cm (IQR, 0.24) and the median right adrenal gland width was 0.75 cm (IQR, 0.18; Table 11).

**Table 11:** Description of the left and right adrenal glands without the dog where one of the adrenal gland was >2.0 cm.

Parameter	*n*	Median	Minimum	Maximum	IQR^1^
Left adrenal gland Cranial pole (cm)	37	0.65	0.39	1.75	0.27
Left adrenal gland Caudal pole (cm)	40	0.69	0.39	1.09	0.23
Difference between left cranial and left caudal pole (cm)	36	0.11	0.0	0.66	0.13
Maximum width left adrenal gland	48	0.70	0.39	1.75	0.24
Right adrenal gland Cranial pole (cm)	37	0.75	0.51	1.3	0.20
Right adrenal gland Caudal pole (cm)	36	0.67	0.30	1.16	0.19
Difference between right cranial and right caudal pole (cm)	30	0.11	0.0	0.72	0.15
Maximum width right adrenal gland	48	0.75	0.50	1.30	0.18
Difference between max. width of left and right adrenal gland (cm)	48	0.13	0.0	0.54	0.15
DVTR	48	1.17	1.00	2.31	0.26
DVTDR	48	0.15	0.0	0.79	0.22

DVTR: dorsoventral thickness difference ratio, DVTDR: dorsoventral thickness difference ratio.

1 IQR: interquartile range.

### Correlation analysis between adrenal gland size, weight, and age

A Spearman correlation analysis identified a significant correlation between adrenal gland size and weight. The maximum width of the left adrenal gland was significantly associated with weight (r = 0.042, 95% CI: 0.1525–0.6344, *p* = 0.002), as was the maximum width of the right adrenal gland (r = 0.339, 95% CI: −0.0557–0.5722, *p* = 0.017). In contrast, there was no significant relationship between maximum adrenal gland size and age. The correlation between the width of the left adrenal gland and age was r = 0.146 (95% CI: −0.1492–0.4175, *p* = 0.316), and was r = 0.097 (95% CI: −0.1971–0.3759, *p* = 0.505) for the right adrenal gland.

It was also investigated whether there was a correlation between adrenal gland size and different weight and age groups. Therefore, the study population were categorized into three weight groups: < 10 kg (n = 17), 10–20 kg (n = 17), and >20 kg (n = 15). The Kruskal-Wallis test revealed a significant difference in the maximum size of the left adrenal gland across these weight groups (*p* = 0.003). A difference was also found for the maximum size of the right adrenal gland, but this did not reach significance (*p* = 0.096). For the analysis of adrenal gland size in relation to age, dogs were divided into two age groups: < 10 years (*n* = 24) and > 10 years (*n* = 25). Using the Mann–Whitney test, there was no correlation between the maximum width of both the left and right adrenal glands and these age groups (*p*-values of 0.254 and 0.681, respectively).

### Comparison of adrenal gland width between dogs with PSP and EP

The maximum width of left and right adrenal gland in dogs with PSP (median 0.76 and 0.79 cm) was not significant different from dogs with an EP (median 0.70 and 0.70 cm; Table 5).

If the dog with one adrenal gland >2.0 cm was excluded from the evaluation, there was also no significant difference between dogs with PSP and dogs with an EP. All data are given in Table 12.

**Table 12:** Comparison of maximum width of the adrenal glands between dogs with partial suppression and escape pattern in dogs without the dog where one of the adrenal gland was >2.0 cm.

Parameter	Escape pattern (*n* = 21)	Partial suppresion pattern (*n* = 27)	*p*
Maximum width of left adrenal gland (cm)	Median: 0.70	Median: 0.75	0.9503
	Range: 0.39–1.19	Range: 0.40–1.75	
	IQR^1^: 0.24	IQR^1^: 0.24	
	(Q^2^_0.75_–Q^2^_0.25_): 0.84–0.60	(Q^2^_0.75_–Q^2^_0.25_): 0.83–0.59	
	*n* = 21	*n* = 27	
Maximum width of right adrenal gland (cm)	Median: 0.70	Median: 0.79	0.6249
	Range: 0.51–1.18	Range: 0.50–1.30	
	IQR^1^: 0.22	IQR^1^: 0.16	
	(Q^2^_0.75_–Q^2^_0.25_): 0.89–0.67	(Q^2^_0.75_–Q^2^_0.25_): 0.85–0.69	
	*n* = 21	*n* = 27	
Difference between max. width of left and right adrenal gland (cm)	Median: 0.15	Median: 0.12	0.5814
	Range: 0–0.54	Range: 0–0.51	
	IQR^1^: 0.13	IQR^1^: 0.21	
	(Q^2^_0.75_–Q^2^_0.25_): 0.18–0.05	(Q^2^_0.75_–Q^2^_0.25_): 0.24–0.03	
	*n* = 21	*n* = 27	
DVTR	Median: 1.17 Range: 1.00–2.31 IQR^1^: 0.24 (Q^2^_0.75_–Q^2^_0.25_): 1.32–1.07 *n* = 21	Median: 1.17 Range: 1.0–1.86 IQR^1^: 0.29 (Q^2^_0.75_–Q^2^_0.25_): 1.33–1.04 *n* = 27	0.7552
DVTDR	Median: 0.15	Median: 0.16	0.7552
	Range: 0–0.79	Range: 0–0.60	
	IQR^1^: 0.20	IQR^1^: 0.25	
	(Q^2^_0.75_–Q^2^_0.25_): 0.27–0.07	(Q^2^_0.75_–Q^2^_0.25_): 0.29–0.04	
	*n* = 21	*n* = 28	

1 IQR: interquartile, CI: confidence interval; DVTR: dorsoventral thickness difference ratio, DVTDR: dorsoventral thickness difference ratio.

### Analysis of factors associated with a specific suppression pattern

Multiple logistic regression analysis was conducted to evaluate the following variables: weight, age, gender, maximum width of the right and left adrenal glands, difference between the maximum widths of the left and right adrenal glands, DVTR, and DVTDR to predict a specific suppression pattern. Choosing EP as the dependent variable, the analysis did not reveal any statistically significant correlations for any of the variables, as all odds ratios had wide confidence intervals including 1, indicating uncertainty in the final conclusion (Supplementary Table S4).

Choosing PSP as the dependent variable or reducing the independent variables to weight, age, gender, maximum width of the right and left adrenal glands, difference between the maximum widths of the left and right adrenal glands did not change results or improve the model.

### Treatment with trilostane

Forty-six dogs were treated with trilostane and showed an improvement in clinical signs. No major adverse events were recorded during treatment. No therapy was initiated in three patients. One of those dogs had epileptic seizures and was euthanized a few days after diagnosis. In the second dog, the owner decided against treatment for financial reasons. In the third dog, the reason for this decision was unknown.

## Discussion

In the present study ultrasonographic data of adrenal glands were retrospectively analyzed in 49 dogs which had a suppression pattern in the LDDST. The study revealed a median maximum width of the left adrenal gland of 0.71 cm and of the right adrenal gland of 0.75 cm.

The LDDST is due to its high sensitivity, the preferred screening test for CS (16, 19, 20, 34). In the present study, the EP was equally frequent as the PSP. This contrasts previous studies in which only 5/24 (20.8%) and 12/42 (28.6%) of dogs with PDH had an EP (20, 21). In the present study, almost all dogs were treated with trilostane, and in none the original diagnosis was questioned due to problems with the treatment. A comparison of adrenal size between dogs with PSP with those having an EP revealed no significant difference in the present study. In two early studies comparing the different patterns of the LDDST in dogs with CS and dogs with non-adrenal illness, the positive predictive value for the EP was 35.7 and 85.7%, respectively. In these studies, an EP was present in 5/59 (8.5%) and in 12/115 (10.4%) of dogs with CS, compared with 9/64 dogs (14.1%) and 2/62 (3.2%) in the non-adrenal illness group. It was concluded that the EP has poor power to predict presence of CS (20, 21). The present study revealed that an EP can reliably diagnose CS. Naturally, other criteria (such as the presence of clinical symptoms) have to be taken into account. In addition, a LDDST should not be performed in any ill dog. Furthermore, a stress-free environment is also important for the accuracy of the test results (23).

This study also demonstrated a mild positive correlation between adrenal gland size and weight. In another study, an association between body weight and age and adrenal gland size was found in dogs with non-adrenal gland insufficiency (27). Correlation between adrenal gland size and age was not observed in this study. The study population consisted of middle-aged to older dogs (median 11 years with a range from 7 to 16 years) with CS, whereas the previously mentioned study had a wider age range with young dogs <4 years, middle age dogs from 4 to 8 years and older dogs over 12 years included (27). Another study found a correlation between age and the width of the left adrenal gland in healthy dogs, whereas there was no correlation with age for the right adrenal gland (35).

This study also examined potential correlations between EP or PSP and adrenal gland size, adrenal asymmetry, weight, age, and gender. The analysis did not identify any statistically significant associations for any of these variables with the LDDS pattern. This finding cannot be discussed in light of the literature as the afore mentioned studies have not performed multiple logistic regression (20, 21).

If all 49 dogs were assessed and the one dog where ADH was suspected was not included, the size of the left adrenals ranged from 0.39 to 1.75 cm and the size of the right adrenals ranged from 0.5 to 1.3 cm, with a median difference of 0.13 cm between both adrenal glands and 0.11 cm within one adrenal gland (cranial and caudal pole).

In the present study, if the dog with a potential ADH is neglected, the maximum diameter of the adrenals was 1.75 cm. If all dogs with DVTDR >0.3 are excluded, the maximum diameter of the adrenals was 1.3 cm. There are no published data to distinguish bilateral adenomas from PDH. Physiologic shape was noticed in 32/38 (84.2%) dogs with images of both adrenal glands in the present study. Maintenance of a physiologic adrenal shape is usually preserved in PDH.

So far, different recommendations regarding the physiologic size of the adrenal glands in dogs exist. A maximum dorsoventral width of the left adrenal greater than 7.4 mm was considered consistent with adrenal enlargement in one study (26). However, other studies showed that the size of the adrenal glands in healthy dogs can vary depending on breed and body size (27, 28). One study, which investigated the association between ultrasonographic adrenal thickness and weight in 266 dogs with non-adrenal gland illness, recommended an adrenal width limit of 0.62 cm for dogs ≤12 kg and of 0.72 cm for dogs >12 kg (27). Another study that examined the adrenal width in healthy dogs from three different weight groups (<10 kg; 10–30 kg; >30 kg) with 15 dogs in each group, and recommended a cut-off of the caudal adrenal pole of ≤0.54 cm for dogs <10 kg, ≤0.68 cm for dogs 10–30 kg, and ≤0.80 cm for dogs >30 kg (36).

Another study proposes separate reference ranges for four different weight groups in dogs with CS (≥2.5–5 kg; >5–10 kg: >10–20 kg; >20–40 kg). The recommended cut-off for the left adrenal gland was 0.51 cm (≥2.5–5 kg), 0.55 cm (>5–10 kg), 0.64 cm (>10–20 kg), and 0.73 cm (>20–40 kg) and for the right adrenal gland 0.53 cm (≥2.5–5 kg), 0.68 cm (>5–10 kg), 0.75 cm (>10–20 kg), and 0.87 cm (>20–40 kg) (29).

Applying the recommendation of Bento et al., 41/48 (85.4%) dogs had an adrenal gland above the cut-off, and 7/48 (14.6%) below the cut-off (27). Similar results were obtained when applying the recommendation of Soulsby et al. to all 42 dogs where the width of at least one caudal adrenal gland was measured. In this case 35/42 (83.3%) dogs had an adrenal gland above the cut-off and 7/42 (16.7%) below the cut-off (36). Applying the recommendation of Melian et al., only 37/48 (77.1%) dogs had adrenal glands above the cut-off and even 11/48 (22.9%) below the cut-off (29) (Supplementary Table S3). The difference compared to the study of Melián et al. could be related to the different inclusion criteria (29). In the present study, CS was diagnosed using the LDDST, which has a higher sensitivity compared to the ACTH stimulation test.

Overall, results of the present study are largely in accordance with these data; however, smaller adrenal glands in a dog suspected to have PDH does not rule out CS. The smallest adrenal gland width in this study, excluding the dog suspected to have ADH, was 0.39 cm of the left adrenal gland and 0.50 cm of the right adrenal gland. CS, in general is considered a progressive disease, where hormonal dysfunction can increase over time (37), and adrenal gland size can be expected to increase over time. However, at the onset of the disease, normal adrenal glands can be present. In addition, some degree of imprecision is expected when assessing abdominal organ size ultrasonographically (25).

In 18.4% (9/49) of cases marked adrenal asymmetry with a DVTDR >0.3 was present. Potentially, in those dogs, ADH was a differential diagnosis. In 5/9 dogs, sonographic reevaluation of the adrenals was performed. All of these five dogs were treated with trilostane, and when sonographic reevaluation took place, the size of both adrenal glands had increased, which is in accordance with the literature that demonstrated an increasing adrenal gland size in dogs treated with trilostane (38, 39). In all 5 dogs a decrease in asymmetry was noted, which argues against the presence of ADH. Myelolipoma was suspected in two of the five dogs due to a hyperechoic round nodule, which also argues against ADH. In one other dog, sonographic reevaluation of the urinary tract was performed, but the adrenals were not mentioned in the report. Images of the adrenals were stored during the initial examination of this dog, and both adrenals had a physiologic shape with a maximum diameter of 0.5 and 0.8 cm, which also argues against presence of ADH. In another dog, imaging of the pituitary gland was performed and a macrotumor was diagnosed. In this dog, maximum adrenal size was 0.54 and 0.99 cm. The cranial pole of the right adrenal was larger (0.99 cm) than the caudal pole (0.74 cm) and was addressed as nodular hyperplasia. Based on these findings, PDH was also very likely in this dog. In two dogs, no follow-up data on adrenal size was available. In one of the two dogs, images of the adrenals were stored, and a physiologic shape was noted. This dog was treated with trilostane for at least 1 year. Despite the presence of asymmetry (width of left adrenal 0.45 cm and width right adrenal gland 0.69 cm), PDH was also very likely in this dog. In the other dog, no saved ultrasonographic images were present, but in the report one adrenal was measured with >2.0 cm (precise width 2.3 cm), whereas the contralateral gland measured 0.3 cm. In this dog, ADH was considered very likely. This dog was also treated with trilostane, but no follow-up information was available. Therefore, 1/49 dog was likely affected by ADH (2%) despite presence of suppression criteria. So, it can be concluded, that PSP and EP of the LDDST are very indicative of PDH, which is in accordance with the literature. Several studies showed an agreement between a LDDST with suppression criteria and PDH (20, 40). Bennaim and colleagues analyzed the pattern of LDDST. All dogs with CS that showed criteria of suppression were diagnosed with PDH, except for one dog. This dog had an adrenal gland >4 cm, but the contralateral adrenal could not be identified and the endogenous ACTH was 31 pg/ml. This dog was thought to have concurrent PDH and ADH (20). It is possible that PDH concurrent with ADH was also present in some of the 9 dogs with adrenal asymmetry in the present study, however, based on the follow-up data this seems unlikely. In the study discussed above, 3/59 dogs (5%) with CS had PDH with concurrent ADH (20). In an older study including 1,500 dogs, 1.1% of the dogs had PDH and ADH concurrently. In this study, histopathological examination of the adrenal and pituitary glands was available in all of the 17 dogs classified as PDH with concurrent ADH. Ten dogs had unilateral cortical adenoma, 4 dogs had bilateral cortical adenoma, and 3 dogs had cortical carcinoma. All 17 dogs had a pituitary neoplasia (microadenoma *n* = 12; macroadenoma *n* = 4; carcinoma *n* = 1). Measurements for the adrenal glands were not reported (31). In a recent study examining 201 dogs with CS, 10 of the dogs (5.0%) had PDH with concurrent pituitary and adrenal lesions. All dogs in this study had a LDDST or a urine-cortisol-creatinine-ratio in combination with oral high-dose dexamethasone suppression test (HDDST) performed to confirm CS. In addition, endogenous ACTH was available in dogs that showed no criteria of suppression in the LDDST or oral HDDST. All dogs underwent CT evaluation of the pituitary and adrenal glands. An enlargement of the pituitary gland was classified as PDH, and an abnormal adrenal gland shape with heterogeneous contrast uptake in combination with increased adrenal thickness > 7 mm was classified as ADH. Two dogs with concurrent adrenal and pituitary lesions were in the dexamethasone-suppressible group (*n* = 78), while the other eight dogs were in the dexamethasone-resistant group. *Post mortem* histopathology was available in one of the two dogs with dexamethasone-suppression and revealed hemangiosarcoma in 1 adrenal gland and a cortical carcinoma in the contralateral adrenal gland (30). Overall, this study also showed that 1.6% of the dogs had PDH and ADH despite being in the dexamethasone-suppressible group. This is in accordance with our results, where one out of 49 dogs (2.0%) very likely did not have PDH despite being dexamethasone-suppressible.

According to an earlier study, equivocal adrenal asymmetry and concurrent width of the smaller of the two adrenals of <0.5 cm is indicative of ADH. However, in the aforementioned study, asymmetry was defined as DVTDR >0.2, and the larger of the two adrenal glands were all >0.7 cm (range, 0.7–8.8 cm) (10). In the present study, we defined adrenal asymmetry as 95% of the upper limit CI for DVTDR >0.3 or DVTR >1.7 since we had more dogs with smaller adrenal glands. Despite this more conservative cut-off, adrenal asymmetry was not uncommon, occurring in 9/49 (18.4%) dogs. In the 49 dogs, the median difference between both adrenal glands was 0.13 cm, indicating approximately uniform adrenal gland width in dogs with PDH. As written previously, one dog can be considered to have had ADH; however, calculation of 95% CI already neglects this one dog. For calculation of the DVTDR, the maximum diameter of the adrenal gland width is advised. In dogs where both poles of the left adrenal gland were recorded (*n* = 37) the median difference between the poles was 0.11 cm, and in dogs where both poles of the right adrenal gland were recorded (*n* = 31) the median was also 0.11 cm, indicating uniform shape of one adrenal gland.

Adrenal asymmetry does not always indicate adrenal neoplasia, as nodular hyperplasia or myelolipoma formation can occur in PDH and cause asymmetrical adrenal glands (10). If the typical shape of the adrenals was maintained, but one of the poles was wider than the other, the term nodular hyperplasia was used in this study. This was noticed in 6/38 dogs (15.8%) and is therefore not an uncommon finding. A myelolipoma was suspected in two dogs of 38 dogs (5.3%) where the adrenal gland images were evaluated, representing therefore an uncommon finding in dogs with PDH. The result corresponds to a study in which 3.8% of the 52 dogs who underwent adrenalectomy had a myelolipoma (18). Precise criteria for the assessment of nodular hyperplasia, myelolipoma, and an adrenal mass in regards to their size, shape and echogenic pattern are largely lacking. One study suggested an adrenal gland more than 4 cm as highly correlating with malignancy (3). In another study of 49 dogs with adrenal tumors comparing adrenal adenomas and adrenal carcinomas, none of the 23 dogs with adrenal adenomas had an adrenal gland >2 cm, whereas 65.4% of the dogs with adrenal carcinomas had an adrenal gland >2 cm. All carcinomas ranged from 0.5 cm to more than 15 cm (41). The majority of the literature proposes consideration of malignancy in adrenals measuring >2 cm (41).

### Limitations

Due to the retrospective nature of the study, there are some limitations in the present study. Measurement of endogenous ACTH and advanced imaging (CT) of the adrenal gland would have been desirable in all dogs with asymmetric adrenal glands. Ultrasonographic measurement of the adrenal gland is subjective to some extent, particularly when performed by different observers. In this study, all ultrasound scans were conducted by highly experienced board-certified Diplomates in Internal Medicine, ensuring a high standard of expertise. However, the potential for observer-dependent variability cannot be entirely excluded. Especially the right adrenal gland is more difficult to image as demonstrated by significant within-day and between-day variation coefficients between three examiners with different levels of expertise (35). None of the dogs in the present study had histopathology to confirm the proposed classification. The limited sample size could also have led to a reduced significance of the study. In particular, because stored images of the adrenal glands were only available from 38 dogs.

## Conclusion

This study showed that all dogs with PSP or EP, except for one case, had adrenal gland size compatible with PDH. In the majority of dogs (85%), adrenal gland width was consistent with the proposed cut-off values; however, this study showed that smaller adrenal gland width does not rule-out PDH. A maximum adrenal gland size >1.7 cm can be considered uncommon with these LDDST patterns. Adrenal asymmetry can occur in up to 18% of dogs. In this group of dogs, measurement of endogenous ACTH and CT imaging of the pituitary and adrenal glands should be advised. Follow up imaging of the adrenals may be indicated in those cases.

## Data Availability

The raw data supporting the conclusions of this article will be made available by the authors, without undue reservation.
