# Supplementary material for: Ultrasonographic adrenal gland changes in dogs with Cushing’s syndrome with a low-dose dexamethasone suppression test result consistent with partial suppression or escape pattern
Source: Front Vet Sci. 2024 Dec 4;11:1477208. doi: 10.3389/fvets.2024.1477208 (PMC11653924; doi:10.3389/fvets.2024.1477208)
Supplement: Supplementary file 1 [file Supplementary_file_1.docx]

Supplementaly Data

TABLE S1 Criteria used to diagnose Cushing`s syndrome in addition to a positive low-dose dexamethasone suppression test

| **Criteria** | **Numbers of dogs** |
| --- | --- |
| 1. Presence of three or more common clinical signs | 35* |
| 2. Presence of at least two common clinical signs and at least one common laboratory abnormality |  |
| 3. Presence of one or more common clinical signs, two or more common laboratory abnormalities, and a positive response to treatment | 10 |
| 4. Presence of at least one common clinical sign, one less common clinical sign, and one common laboratory abnormality | 3 |
| 5. Presence of at least one common clinical sign and at least four common laboratory abnormalities | 1 |

* in 19/35 dogs, criteria 1 and 2, in 2/35 criteria 1, and in 14/35 dogs criteria 2 applied.

TABLE S2 Overview of clinical and laboratory diagnostic findings of all 49 dogs

| **Dog** | **Polyuria/Polydipsia** | **Polyphagia** | **Increased panting** | **Abdominal distension** | **Muscle weakness/-atrophy** | **Endocrine alopecia** | **Calcinosis cutis** | **Hyperpigmentation** | **Lethargy/exercise intolerance** | **Insulin-resistant Diabetes mellitus** | **AP ↑** | **ALT ↑** | **Cholesterol ↑** | **Neutrophilia and/or Lymphopenia** | **Thrombocytosis** | **USG < 1.020** | **Proteinuria (UPC > 1)** | **UCC ↑** | **ACTH stimulation test diagnostic** | **Positive response to treatment** |
| --- | --- | --- | --- | --- | --- | --- | --- | --- | --- | --- | --- | --- | --- | --- | --- | --- | --- | --- | --- | --- |
| 8 y, f, Jack Russell Terrier | + | + |  | + |  | + |  |  |  | - | - | + |  | + | - | + |  |  | + | + |
| 12 y, f/s, mixed breed | + | + | + |  |  |  |  |  |  | - |  |  |  |  |  |  |  | + | - |  |
| 9 y, f/s, West Highland White Terrier | + |  |  |  |  | + |  | + |  | - | + |  |  |  |  |  |  |  | - | + |
| 11 y, f/s, Pomeranian | + |  | + | + |  |  |  |  |  | - | + |  |  | + | + | - |  | + | - | + |
| 9 y, m, Dachshund | + |  |  | + |  | + |  |  |  | - | + | + | + | + | + | + |  |  | - | + |
| 13 Data y, m/n, Dachshund |  |  |  | + |  |  |  |  |  | - | + | + | - | + | + | - |  |  | - |  |
| 15 y, f/s, Anglo-Français de petite vénerie | + |  |  |  |  | + |  |  |  | - | + |  |  | + | - | + |  |  | - |  |
| 12 y, f/s, West Highland White Terrier | - | - | + |  |  |  |  |  |  | - | + |  |  | + | + | + |  |  | - | + |
| 10 y, m/n, West Highland White Terrier | + |  |  | + |  | + |  | + |  | - | - |  |  | - | + | + |  |  | - | + |
| 9 y, m, Jack Russell Terrier | + | + |  |  |  | + |  |  |  | - | + | + |  | + | - | + | + |  | - | + |
| 9 y, m, Bichon Frisé |  |  |  |  |  | + | - | + |  | - | + |  |  |  |  |  |  | + | - | + |
| 8 y, m/n, Dalmatian | - | - |  | + |  | - | - | - |  | - | + |  | + | - | - | - |  |  | - | + |
| 9 y, f/s, West Highland White Terrier | + |  | + | + |  | + | - | + |  | - | - |  | - |  |  | + |  | + | - | + |
| 13 y, m, mixed breed | + | + |  | + |  |  |  |  |  | - | + | + |  |  | + | - |  |  | - |  |
| 11 y, f/s, Dalmatian | + |  |  |  |  |  | - | - |  | + | + |  |  |  | - | - |  |  | + | + |
| 8 y, m, Yorkshire Terrier | + | + | + |  | + |  |  |  |  | - | - |  | - | - | - | + |  |  | - | + |
| 16 y, m/n, Yorkshire Terrier | + |  | + |  |  |  | - | + |  | - | + |  |  |  | - | + | - |  | + | + |
| 11 y, m, Yorkshire Terrier | - |  | + | + |  |  |  |  |  | - | - |  | - | - | + | - | + |  | + | + |
| 14 y, m/n, Cairn Terrier | + | + |  |  |  | + |  |  |  | - | - |  | - | + | - | - |  |  | + | + |
| 13 y, f/s, Magyar Viszlar | + |  |  |  |  |  |  |  |  | - | + | + |  | - | - | - |  |  | - | + |
| 10 y, f, Golden Retriever | + | + |  |  |  | - | - | + |  | - | + | + |  | - | - | + |  | + | - | + |
| 12 y, f, Labrador Retriever | + |  |  | + |  | + | - | + |  | - | + | + |  | + | - | - |  |  | + | + |
| 9 y, f/s, mixed breed | + | - |  |  |  |  |  |  |  | - | + | + |  | + | + | + |  |  | - | + |
| 11 y, f/s, mixed breed | + | + |  | + | + |  |  |  |  | - | + | + |  | - | - | + | + |  | - | + |
| 8 y, f, Yorkshire Terrier | + | + | + | + |  | + | - | + |  | - | - |  |  | - | + | + |  |  | - | + |
| 9 y, f/s, Rhodesian Ridgeback | + | + | + |  | + |  | + |  | + | - | + | + |  | + | - | + |  | + | - | + |
| 11 y, m/n, mixed breed | + | + |  |  |  |  |  |  |  | - | + | + | + | + | - | - |  | + | - | + |
| 13 y, m/n, West Highland White Terrier | + | + | + | + |  |  |  |  |  | - | - |  |  | - | + | - |  |  | - | + |
| 8 y, f/s, mixed breed | + | + |  |  |  |  |  | + |  | - | + |  |  | - | + | + | + |  | - | + |
| 11 y, m, English Cocker Spaniel | + | - |  |  |  |  |  |  |  | + | + | + |  | - | + | - |  |  | - | + |
| 8 y, f, mixed breed | - | + | + |  |  |  |  |  | + | + | + |  |  |  | - | - |  |  | - |  |
| 11 y, f, mixed breed | + |  |  |  |  |  |  | + |  | - | + |  | - | - | + | + | + |  | - | + |
| 13 y, f/s, West Highland White Terrier | - |  |  | + |  | - | - | + |  | - | + |  |  | + | - | + |  |  | - | + |
| 13 y, f/s, mixed breed | + | - |  |  |  |  |  |  |  | - | + | + |  | - | - | + |  |  | - | + |
| 10 y, m/n, Labrador Retriever | + | + |  |  |  | + | - | - |  | - | + |  |  | - | - | + |  |  | - | + |
| 12 y, f, mixed breed | - | + |  |  |  |  |  |  |  | - | + | + |  | - | - | - |  |  | - | + |
| 14 y, m/n, mixed breed | + | + |  | + |  |  |  |  |  | - | + |  | - | - | - | - |  |  | - | + |
| 10 y, f/s, Cavalier King Charles Spaniel | + |  |  |  |  | + |  |  |  | - | + |  |  | + | + | + |  | + | - | + |
| 10 y, f/s, mixed breed | + | - |  |  |  | + |  |  |  | - |  |  | - | + | - | - |  |  | - | + |
| 12 y, f, Bearded Collie | + |  |  |  |  | + |  | + |  | - | + |  |  | - | + | + |  | + | - | + |
| 12 y, m/n, mixed breed | - | - | + |  |  |  |  |  |  | - | + | + |  | - | - | - |  |  | - | + |
| 8 y, f/s, Magyar Viszlar | + | + |  |  |  |  |  |  |  | - | + |  | - | - | - | + |  |  | - | + |
| 10 y, f/s, Australian Labradoodle | + | + | + | + | + | - | - |  |  | - |  |  |  | - | + | + |  |  | - | + |
| 16 y, m/n, Jack Russell Terrier | + |  |  |  |  |  |  |  | + | - | + |  | - | - | - | - | - |  | - | + |
| 9 y, m, Yorkshire Terrier | + |  |  |  |  |  |  |  |  | - |  |  |  | - | + | + | + | + | - | + |
| 7 y, f, Parson Russel Terrier | + | + |  | + |  |  |  |  | + | - | + | + | + | - | - | - | + |  | - | + |
| 7 y, f/s, French Bulldog | + | + | + | + | + | + | + |  | + | - |  |  | - | - | - | - |  |  | - | + |
| 9 y, f/s, Jack Russell Terrier | + | + |  | + |  |  |  |  |  | - | + | + |  | + | - | - | + | + | - | + |
| 12 y, f/s, mixed breed | - | + |  | - |  | - | - | - |  | - |  |  |  | + | - | + |  |  | - | + |

Symbols: + indicates the presence of a symptom or finding, - indicates the absence of a symptom or finding, If neither symbol is present, no data was available for this parameter; ACTH stimulation test diagnostic +: 1h-Cortisolspiegel> 20 µg/dl

ALT ↑: elevated alanine aminotransferase; AP ↑: elevated alkaline phosphatase; Cholesterol ↑: elevated cholesterol levels; f: female; f/s: female spayed; LDDST: low-dosedexamethasone suppression test; m: male; m/n: male neutered; UCC ↑: elevated urine cortisol to creatinine ratio; UP/C: Urinary Protein-to-Creatinine Ratio; USG: Urine specific gravity, y: years

TABLE S3 Applying different recommendation of an adrenal width limit for the left and right adrenal according to the recommendations by Bento et al. 2016, Soulsby et al. 2015, and Melian et al. 2021, to this study population excluding the dog, who was suspected to have ADH.

|  | **Cut-off adrenal pole*** | |  | **n = 48** |
| --- | --- | --- | --- | --- |
| **≤ 12 kg** | < 0.62 cm | |  | n = 3 |
|  | > 0.62 cm | |  | n = 24 |
| **> 12 kg** | < 0.72 cm | |  | n = 4 |
|  | > 0.72 cm | |  | n = 17 |
|  | **Cut-off caudal adrenal pole**** | |  | **n = 42** |
| **< 10 kg** | ≤ 0.54 cm | |  | n = 1 |
|  | > 0.54 cm | |  | n = 14 |
| **10–30 kg** | ≤ 0.68 cm | |  | n = 4 |
|  | > 0.68 cm | |  | n = 17 |
| **> 30 kg** | ≤ 0.80 cm | |  | n = 2 |
|  | > 0.80 cm | |  | n = 4 |
|  | **Cut-off adrenal gland ***** | |  | **n = 48** |
|  | **Left adrenal gland** | **Right adrenal gland** |  |  |
| **≥ 2.5–5 kg** | ≤ 0.51 cm | ≤ 0.53 cm |  | n = 0 |
|  | > 0.51 cm | > 0.53 cm |  | n = 3 |
| **> 5–10 kg** | ≤ 0.55 cm | ≤ 0.68 cm |  | n = 4 |
|  | > 0.55 cm | > 0.68 cm |  | n = 11 |
| **> 10–20 kg** | ≤ 0.64 cm | ≤ 0.75 cm |  | n = 3 |
|  | > 0.64 cm | > 0.75 cm |  | n = 13 |
| **> 20–40 kg** | ≤ 0.73 cm | ≤ 0.87 cm |  | n = 4 |
|  | > 0.73 cm | > 0.87 cm |  | n = 10 |

*Bento et al. 2016, ** Soulsby et al. 2015, *** Melian et al. 2021, n = number

**TABLE S4** Multiple Regression Analysis to identify correlation of any of the variables are associated with Escape pattern

| **Variable** | **Odds Ratio (OR)** | **95% CI OR** |
| --- | --- | --- |
| **Intercept (β0)** | 0.2469 | 2.75e-05 – 11059 |
| **Weight (β1)** | 1.009 | 0.9581 – 1.066 |
| **Age (β2)** | 1.146 | 0.8549 – 1.564 |
| **Maximum width left adrenal gland (β3)** | 0.7069 | 0.002416 – 227.1 |
| **Maximum width right adrenal gland (β4)** | 0.9201 | 0.008868 – 96.98 |
| **Difference between max. width of left and right adrenal gland (β5)** | 0.93027 | 2.384e-04 – 1.03e+08 |
| **DVTR (β6)** | 0.7848 | 5.636e-005 – 68.29 |
| **DVTRDR (β7)** | 29.72 | 7.203e-007 – 5.329e10 |
| **Gender (male) (β8)** | 1.104 | 0.2980 –4.014 |

Tjur's R² = 0.0475. ROC AUC = 0.6173. 95% CI (ROC) = 0.4569 – 0.7778

DVTR: dorsoventral thickness difference ratio, DVTDR: dorsoventral thickness difference ratio
